# Ionic Hydrogel Triboelectric Sensor for Real‐Time Swimming Kinematics Monitoring and Stroke Recognition

**DOI:** 10.1002/open.70208

**Published:** 2026-04-20

**Authors:** Xiao Liang, Zhao Liu

**Affiliations:** ^1^ Sports department Capital University of Economics and Business Beijing China; ^2^ Sport department Communication University of China Beijing China

**Keywords:** hydrogel electrode, intelligent sports, self‐powered sensor, swimming monitoring, triboelectric nanogenerators

## Abstract

Recently, wearable sports electronics have attracted increasing attention for their potential to enable continuous motion tracking and energy‐autonomous operation in complex athletic environments. In this work, we present a flexible triboelectric nanogenerator (PAC‐TENG) constructed from a PVA/Ala/CaCl_2_ ionic hydrogel electrode and a compliant silicone triboelectric layer. The PAC hydrogel provides high stretchability, stable ionic conductivity, and strong deformation tolerance, while the silicone surface ensures efficient and repeatable charge generation. Benefiting from this synergistic design, the PAC‐TENG achieves excellent electrical outputs, including a peak voltage of 472.9 V, a short‐circuit current of 37.1 µA, and a transferred charge of 104.1 nC, along with high power density under matched load. The device exhibits strong sensitivity to bending angle, applied force, and motion frequency, enabling accurate perception of human biomechanics. When mounted on swimming‐related joints, the PAC‐TENG can reliably monitor stroke patterns and detect abnormal motion signals, highlighting its promise for self‐powered aquatic sports analytics and intelligent safety monitoring.

## Graphical abstract

A flexible self‐powered triboelectric nanogenerator (TENG) based on PVA/Ala/CaCl2 hydrogel is developed for motion sensing. The device exhibits stable electrical output and good mechanical properties. It enables real‐time monitoring of human joint movements and demonstrates potential for swimming motion monitoring and wearable smart sports applications.

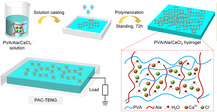

## 1 Introduction

Recently, the growing dependence of modern society on conventional fossil fuels has accelerated the depletion of exploitable reserves, while rapid industrialization and urban expansion continue to intensify global environmental pressures [1, 2, 3]. To address these challenges, nations worldwide are actively restructuring their energy systems by improving energy‐use efficiency and advancing renewable energy technologies [4, 5, 6]. Meanwhile, the emergence of intelligent devices and the rapid evolution of next‐generation sports technology have created an urgent demand for safe, reliable, and sustainable power sources for smart wearable electronics [7, 8, 9, 10, 11]. In the intelligent sports applications, such as athlete posture tracking, real‐time physiological state assessment, immersive motion‐capture training, and environmental‐adaptive safety monitoring in underwater or outdoor scenarios, require miniaturized, flexible, and continuously operating self‐powered sensing systems [12, 13]. Since 2012, triboelectric nanogenerators (TENGs) have attracted significant attention as an ideal technology for constructing self‐powered intelligent motion sensors, owing to their highly tunable structural configurations, broad material compatibility, and intrinsic capability to directly harvest energy from mechanical motions [14, 15, 16, 17, 18, 19, 20, 21, 22, 23, 24, 25, 26, 27, 28, 29, 30, 31, 32, 33, 34, 35, 36]. By converting diverse forms of biomechanical or environmental energy—including joint bending, muscle vibrations, impact forces during running, wind flow, and water movement during swimming—into electrical signals, TENGs provide a fundamentally self‐sustained energy platform for advanced motion monitoring, posture recognition, endurance evaluation, and high‐precision sport‐tech analytics [18, 37, 38, 39, 40, 41, 42, 43, 44, 45, 46, 47, 48, 49]. However, the growing demand for TENGs in intelligent sports scenarios highlights the need for materials capable of withstanding large deformations, frequent mechanical cycling, and complex outdoor or aquatic environments, all while maintaining biocompatibility and sustainability [50, 51]. As a result, flexible, eco‐friendly, and skin‐adaptive materials have become essential for enabling the next wave of high‐performance self‐powered sports sensors [52]. Biodegradable polymers and ionic hydrogels, with their softness, tunable mechanics, biocompatibility, and sustainability, are promising materials for green TENGs and enable new opportunities in intelligent sports systems, wearable health monitoring, athletic safety, and IoT‐based motion analytics [53].

With the rapid evolution of flexible electronics and intelligent motion‐monitoring technologies, there is a growing demand for triboelectric materials that are self‐powered, highly stretchable, and environmentally adaptive [54]. Although various degradable polymers have been introduced into green TENG systems in recent years, conventional degradable films often exhibit limited mechanical flexibility, insufficient wear resistance, and poor tolerance to large deformation, restricting their advancement toward softness, miniaturization, and complex‐motion adaptability [55]. Hydrogels, benefiting from their biphasic solid–liquid architecture, outstanding stretchability, softness, bio‐safety, and tunable ionic conductivity, have emerged as more promising candidates for constructing deformable and biodegradable triboelectric layers [56, 57]. Among hydrogel matrices, polyvinyl alcohol (PVA) is widely used due to its low cost, biocompatibility, and abundant hydroxyl groups capable of forming diverse weak interactions that stabilize a three‐dimensional polymer network [58]. Nonetheless, a single‐network PVA hydrogel rarely satisfies the concurrent requirements of high mechanical robustness, efficient ion transport, and structural stability demanded in high‐strain wearable applications [59]. To address this challenge, recent strategies have focused on incorporating secondary molecular phases or functional ionic species to form synergistic multinetwork structures [60]. Small molecules with coordination‐active groups or ions capable of regulating hydration states can reinforce dynamic cross‐linking, enhance deformation tolerance, and improve frost resistance by modulating the balance between free and bound water [61]. Meanwhile, inorganic salts have gained particular attention for simultaneously boosting ionic conductivity and imparting reliable anti‐freezing capability in a simple, low cost, and biocompatible manner. While conductive fillers, such as graphene [62], carbon nanotube [63], MXene [64], polyaniline [65], and polydopamine [66], can further increase conductivity, they often compromise transparency and mechanical softness. Therefore, constructing highly transparent, mechanically resilient, ionically conductive, and biodegradable hydrogels through multiphase cooperative networks combined with inorganic‐salt‐assisted ion transport has become an effective materials design pathway, providing a solid foundation for next‐generation flexible, self‐powered TENGs capable of operating reliably in complex motion environments [67, 68, 69, 70, 71].

In this work, we developed a flexible triboelectric nanogenerator (PAC‐TENG) that integrates a PVA/Ala/CaCl_2_ ionic hydrogel electrode with a highly compliant silicone triboelectric layer, aiming to achieve efficient biomechanical‐energy harvesting and intelligent motion sensing under complex sports environments, including swimming, running, and multijoint training scenarios. The PAC hydrogel exhibits remarkable mechanical stretchability, robust ionic conductivity, and stable coordination‐assisted network integrity, while maintaining excellent softness and conformability, making it an ideal candidate for deformable self‐powered electrodes. Meanwhile, the silicone layer provides a smooth, durable, and strongly electron‐accepting surface, ensuring consistent triboelectric charge generation during repeated contact‐separation cycles. Benefiting from this synergistic structural design, the PAC‐TENG delivers outstanding electrical performance, achieving a peak open‐circuit voltage of 472.9 V, a short‐circuit current of 37.1 μA, and a maximum transferred charge of 104.1 nC. The PAC‐TENG device also demonstrates a maximum output power of 1.27 mW under matched load conditions, confirming its strong energy‐conversion efficiency for practical self‐powered applications. Moreover, the electrical output is highly sensitive to variations in mechanical parameters, such as bending angle, applied force, frequency, and separation distance, enabling precise perception of human motion intensity and joint kinematics. When attached to swimming‐relevant joints, such as the finger, wrist, and elbow, the PAC‐TENG can continuously track stroke patterns and force output, and it is also capable of identifying abnormal behaviors that may indicate potential danger during underwater motion. These results demonstrate the PAC‐TENG as a promising platform for next‐generation self‐powered aquatic sports analytics, wearable health monitoring, and intelligent safety alert systems.

## 2 Experiments

### 2.1 Materials

Poly(vinyl alcohol) (PVA, 1799 grade, 98–99% hydrolyzed) was bought from Chengdu Blue Whale Technology Co., Ltd, China. L‐alanine (Ala) was purchased from Hebei Rencan Biotechnology Co., Ltd, China. Calcium chloride (CaCl_2_, anhydrous) was obtained from Shandong Hezhan Chemical Co., Ltd, China. Nylon films (thickness: 50–80 μm) were used as the flexible supporting substrate and the positive triboelectric layer, which was bought from Kunshan Meichuang Precision Machinery Co., Ltd, China. The two‐part silicone elastomer (components A and B, purchased from Foshan Nanhai Hefeng Rubber Products Co., Ltd, China) was used as the silicone layer. Deionized water, produced by a Milli‐Q purification system (Suzhou Bitian Purification Equipment Co., Ltd, China), was used throughout the preparation of hydrogels. Copper foil and conductive copper wires were employed as electrodes for the TENG device. All materials were handled at ambient laboratory conditions.

### 2.2 The Preparation Process of PVA/Ala/CaCl_2_ Hydrogel

PVA/Ala/CaCl_2_ hydrogels were prepared by a simple one‐pot solution casting method. Briefly, 2.5 g of PVA was dispersed in 40 mL of deionized water and heated to 95°C under magnetic stirring for 4 h until a clear solution was obtained, followed by 30 min of ultrasonication to remove bubbles and ensure homogeneity. In parallel, 1.5 g of Ala was dissolved in a small amount of deionized water at 80°C with stirring for 2 h. The Ala solution was then slowly added into the hot PVA solution under vigorous stirring, and the mixture was kept at 80°C for an additional 1 h to promote sufficient interaction between PVA and Ala chains. Subsequently, different amounts of CaCl_2_ (e.g., 0.5, 1.0, and 1.5 g) were introduced into the PVA/Ala mixture, followed by 1 h of stirring and 30 min of ultrasonication to obtain a uniform precursor solution. The final solution was poured into PTFE molds and allowed to gel at room temperature (≈25°C) for 48–72 h to form bulk PVA/Ala/CaCl_2_ hydrogels. The samples with increasing CaCl_2_ contents were denoted as PAC‐1 (0.5 g CaCl_2_), PAC‐2 (1 g CaCl_2_), and PAC‐3 (1.5 g CaCl_2_), respectively, and were cut into the desired shapes for subsequent characterization and TENG fabrication. Room‐temperature gelation (≈25°C) was selected as a mild and practical condition to avoid additional thermal effects on the PVA‐based network. The gelation period of 48–72 h was chosen to allow sufficient network stabilization while minimizing excessive moisture evaporation during prolonged standing.

### 2.3 The Preparation Process of PAC‐TENG Device

The PAC‐TENG device was constructed using the PVA/Ala/CaCl_2_ hydrogel as the ionic electrode and a silicone elastomer as the triboelectric layer. First, the hydrogel electrode was cut into rectangular films (40 × 20 × 1 mm). The triboelectric layer was prepared by mixing silicone component A and component B (purchased from China) at a mass ratio of 1:1. After vigorous stirring for 3 min to ensure uniform cross‐linking, the mixture was poured into a PTFE mold and cured at room temperature for 12 h to obtain a compliant silicone rubber film with excellent flexibility and strong electron‐accepting capability. During assembly, the cured silicone rubber was used to fully encapsulate the hydrogel electrode, forming a conformal contact interface that enhances structural robustness and ensures stable contact‐separation motion. The encapsulated hydrogel electrodes were finally fixed on two acrylic substrates with a 10‐mm spacer at each corner, leading to the formation of a complete PAC‐TENG in vertical contact‐separation mode.

### 2.4 Characterization and Measurements

The microstructure and surface morphology of the PVA/Ala/CaCl_2_ hydrogel electrodes and silicone triboelectric layer were examined using scanning electron microscopy (SEM), providing insight into the internal porous network and the interfacial characteristics relevant to charge generation. The ionic conductivity of the hydrogel was quantified via electrochemical impedance spectroscopy (EIS) using a two‐electrode configuration, and conductivity values were calculated according to *σ* = L/(R·S). The electrical characteristics of the PAC‐TENG, including *V*_OC_, *I*_SC_, and *Q*_SC_, were measured using a high‐precision electrometer (Keithley 6514, USA). During testing, the PAC‐TENG device was subjected to well‐defined periodic contact‐separation cycles generated by a programable vibration platform, which ensured accurate control over the applied force, displacement, and operating frequency. This controlled mechanical excitation allowed the acquisition of stable and repeatable output signals, thereby guaranteeing the reliability and reproducibility of the measured triboelectric performance.

## 3 Results and Discussion

### 3.1 Structural Design and Fabrication Overview of the PAC‐TENG

**FIGURE 1:**
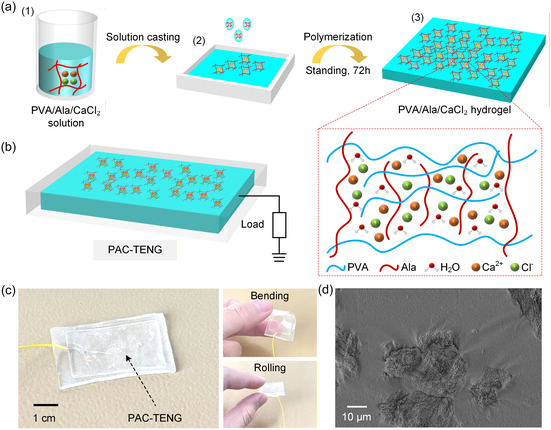
**Schematic illustration and structural characterization of the PAC‐TENG.** (a) Fabrication process of the PVA/Ala/CaCl_2_ (PAC) hydrogel. (a1) Preparation of the precursor solution containing PVA, Ala, and CaCl_2_. (a2) Solution casting of the homogeneous mixture into a flat mold. (a3) Polymerization and network formation after standing for 72 h. (b) Structural design of the PAC‐TENG device. (c) Optical images of the assembled PAC‐TENG device and its excellent mechanical deformability. (d) SEM image of the freeze‐dried PAC hydrogel surface.

Figure 1a1 shows the initial formation of the hydrogel precursor. Specifically, 2.5 g of PVA was dissolved in 40 mL deionized water at 95°C for 4 h, followed by 30 min ultrasonication. Then, 1.5 g of Ala was dissolved separately at 80°C for 2 h and introduced into the PVA solution. Subsequently, different amounts of CaCl_2_ (0.5, 1.0, and 1.5 g) were added, followed by 1 h stirring and 30 min ultrasonication to obtain a homogeneous precursor solution. As illustrated in Figure 1a2, the precursor was then cast into a PTFE mold to form a uniform layer. After gelation at room temperature for 48–72 h, transparent and mechanically robust PVA/Ala/CaCl_2_ hydrogels were obtained, as shown in Figure 1a3. The samples with increasing CaCl_2_ contents were denoted as PAC‐1, PAC‐2, and PAC‐3, respectively. The synergistic interactions—including hydrogen bonding, ionic coordination, and electrostatic association—contribute to the hydrogel's enhanced mechanical integrity and ion transport capability. The overall structure of the PAC‐TENG is illustrated in Figure 1b, where the hydrogel layer serves as a compliant and conductive electrode. The device operates in a vertical contact‐separation mode, with the hydrogel encapsulated by a silicone‐based triboelectric layer to form an integrated unit. Upon mechanical excitation, the periodic contact between the silicone surface and an external frictional counterpart induces charge transfer, while the hydrogel electrode collects and transmits the induced electrical signals to the external load. This configuration enables stable electrical output under various deformation modes owing to the hydrogel's softness and excellent conformability. The practical flexibility of the fabricated PAC‐TENG is demonstrated in Figure 1c. The device maintains structural integrity under bending, folding, and rolling, confirming strong mechanical robustness and suitability for wearable or deformable electronics. The thin and transparent silicone encapsulation allows the hydrogel electrode to remain well‐protected while preserving its ability to deform without fracture. Figure 1d presents the SEM image of the hydrogel microstructure. The surface exhibits interconnected domains and textured regions, reflecting the heterogeneous yet continuous polymer–ion network formed by PVA chains and Ala molecules coordinated with Ca^2+^. This microstructure supports efficient charge transport and provides abundant ion–water interaction sites, which are essential for achieving stable and high‐output triboelectric performance.

### 3.2 Working Mechanism of the PAC‐TENG Device

**FIGURE 2:**
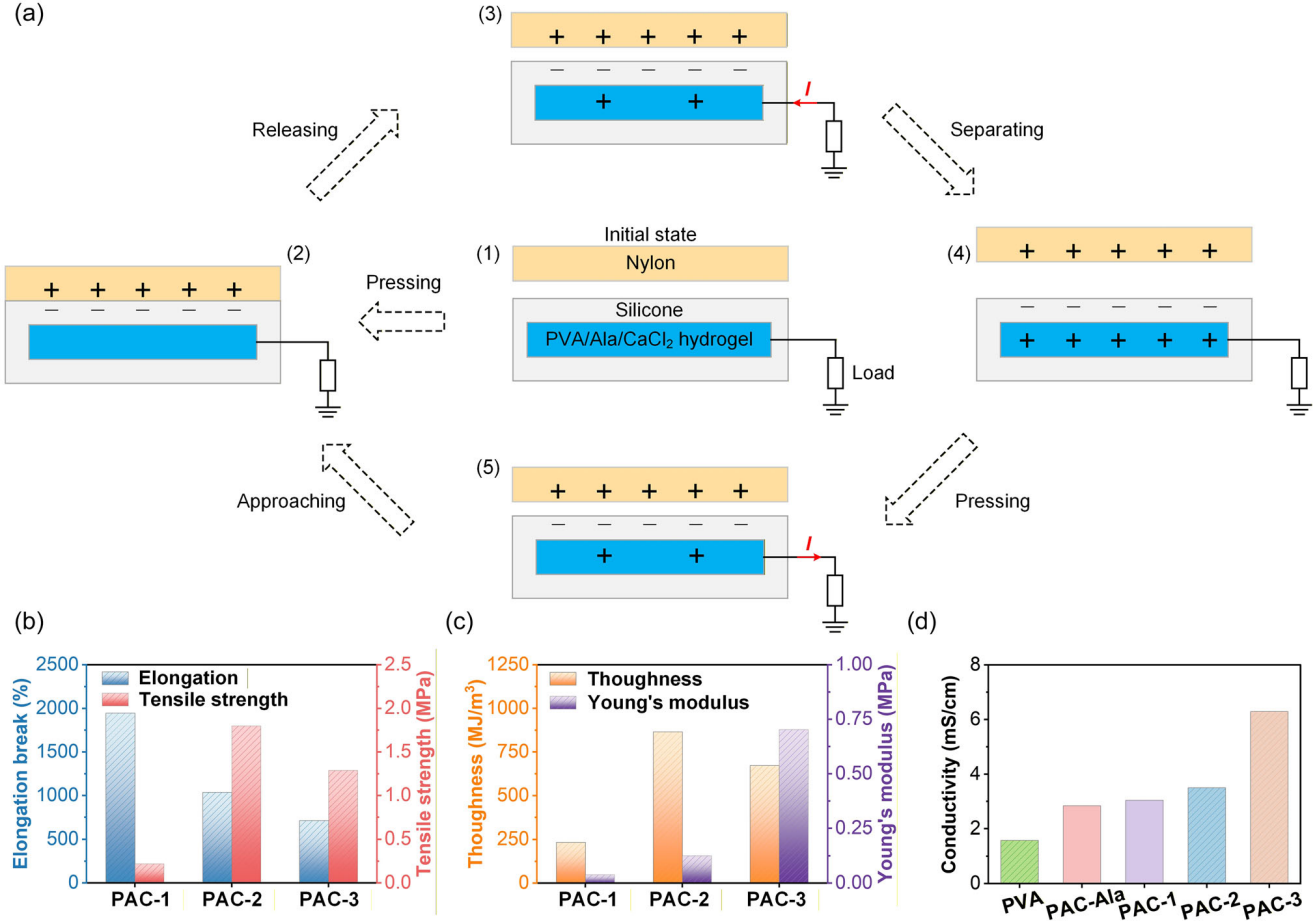
**Overall working mechanism and fundamental properties of the PAC‐TENG.** (a1‐a5) Schematic illustrations of the contact‐separation operation cycle of the PAC‐TENG. (b) Elongation at break and tensile strength of PAC hydrogels with different CaCl_2_ contents (PAC‐1, PAC‐2, PAC‐3). (c) Toughness and Young's modulus of PAC hydrogels. (d) Ionic conductivity of pristine PVA, PVA/Ala hydrogel, and PAC hydrogels (PAC‐1, PAC‐2, PAC‐3).

Figure 2a1 illustrates the initial state of the PAC‐TENG device, in which the nylon film (negative triboelectric layer) and the silicone‐encapsulated PVA/Ala/CaCl_2_ hydrogel electrode (positive triboelectric layer) remain fully separated with no external force applied. In this condition, no charge transfer occurs, and the system maintains an electrostatic equilibrium, thereby producing no output signal. Figure 2a2 demonstrates the approaching and pressing process. When an external force drives the nylon layer into intimate contact with the silicone surface, triboelectric charges are generated due to the contact electrification effect. Owing to the large difference in their triboelectric polarities, the nylon surface gains electrons and becomes negatively charged, whereas the silicone surface loses electrons and becomes positively charged. As a result, equal but opposite triboelectric charges accumulate at the interface. Because the hydrogel electrode is underneath the silicone layer, electrostatic induction causes electrons within the conductive hydrogel to redistribute correspondingly, establishing an induced potential difference across the external circuit. Figure 2a3 depicts the releasing process, during which the external pressure is removed, and the two triboelectric layers begin to separate. As the gap gradually widens, the electrostatic field between the oppositely charged surfaces increases. This induces electron flow from the hydrogel electrode toward the ground through the external load, generating a transient current. The magnitude of the induced current is governed by the separation rate and the preserved triboelectric charge density. Figure 2a4 shows the fully separated state. At maximum separation, the electrostatic induction reaches equilibrium, and no further electron flow occurs. The device remains in a highly polarized state, with the nylon surface retaining negative charges and the silicone side remaining positively charged. The output signal stabilizes at this moment. Figure 2a5 presents the subsequent repressing stage, where the two surfaces move toward each other again. As the distance decreases, the electrostatic potential difference collapses, driving electrons to flow back in the opposite direction through the external circuit. This reverse current completes a full contact‐separation cycle, resulting in the characteristic alternating‐current output of the PAC‐TENG device.

### 3.3 Comprehensive Mechanical and Electrical Characterization of PAC Hydrogels

Figure 2b presents the mechanical performance of PAC hydrogels with varying CaCl_2_ loadings (PAC‐1, PAC‐2, and PAC‐3), demonstrating the substantial influence of ionic cross‐linking density on their tensile properties. As the Ca^2+^ concentration increases, both the elongation at break and tensile strength exhibit notable improvements. PAC‐1 shows moderate extensibility with limited tensile strength due to its relatively loose polymer–ion network. In contrast, PAC‐2 achieves the highest elongation (>2000%) and significantly enhanced tensile strength, indicating the formation of a more robust dual‐network structure composed of PVA crystalline regions and Ca^2+^‐mediated coordination bonds. Further increasing the CaCl_2_ content to form PAC‐3 results in a slight decrease in elongation but a continuous rise in strength, suggesting that excessive ionic content increases network stiffness and reduces chain mobility. These trends confirm that the synergistic interactions among PVA chains, alanine molecules, and Ca^2+^ ions effectively tune the mechanical resilience of the hydrogels. The corresponding toughness and Young's modulus, depicted in Figure 2c, further highlight the strengthening effects of increasing Ca^2+^ concentration. PAC‐2 exhibits the highest toughness, reflecting its optimal balance between stretchability and cross‐linking density, whereas PAC‐3 displays the highest modulus, consistent with a more rigid ionic network. These results collectively demonstrate that the mechanical behavior of PAC hydrogels can be precisely modulated by adjusting calcium‐ion content, enabling their integration as durable and compliant electrodes in flexible TENG devices. Finally, Figure 2d compares the ionic conductivity of PVA, PVA/Ala, and PAC hydrogels. The incorporation of alanine moderately enhances conductivity due to additional hydrogen‐bonding interactions and zwitterionic groups. The introduction of CaCl_2_ leads to a marked increase in ionic conductivity as Ca^2+^ and Cl^−^ ions establish continuous ion‐transport pathways within the hydrogel matrix. PAC‐3 exhibits the highest conductivity, validating the significant contribution of free mobile ions. The combined high mechanical robustness and ionic conductivity underline the suitability of PAC hydrogels as efficient electrodes for high‐performance PAC‐TENGs.

### 3.4 Influence of Operating Frequency and Calcium Ion Concentration on the Electrical Output Performance of PAC‐TENG Device.

**FIGURE 3:**
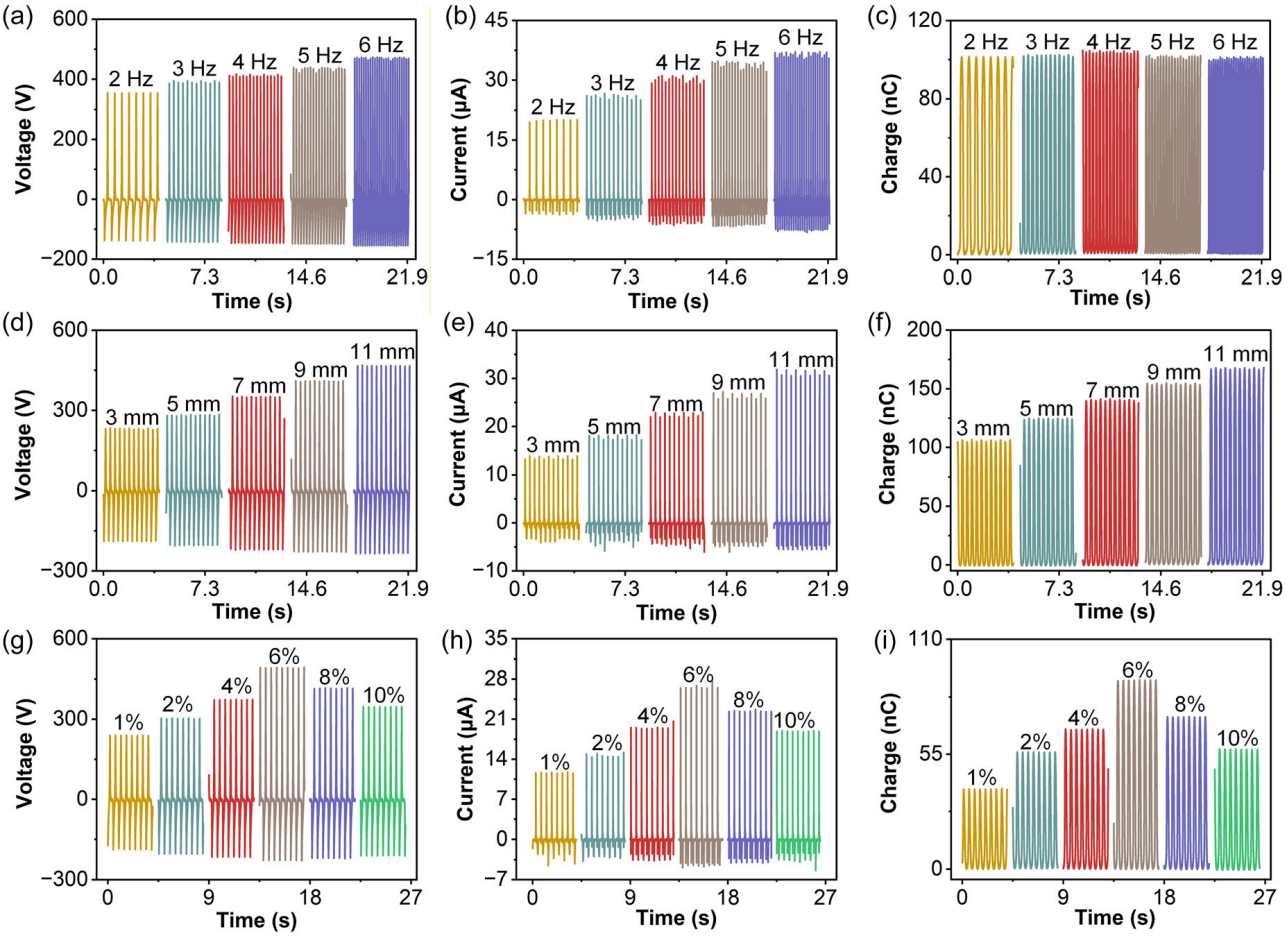
**Output performance of the PAC‐TENG under different operating conditions.** (a–c) Electrical output responses of the PAC‐TENG measured under various operating frequencies. (d–f) Output characteristics of the PAC‐TENG at different separation distances. (g–i) Influence of CaCl_2_ concentration on the electrical performance of the PAC‐TENG.

In Figure 3a, the *V*_OC_ is recorded at excitation frequencies ranging from 2 to 6 Hz under a constant contact force and separation distance. The voltage waveforms remain stable and periodic across all frequencies, while the peak *V*_OC_ increases from approximately 353.4 V at 2 Hz to 472.9 V at 6 Hz, reflecting faster charge redistribution and reduced time for charge recombination during each contact‐separation cycle. Figure 3b presents the corresponding *I*_SC_ responses, where the peak *I*_SC_ rises significantly—from around 20.1 μA at 2 Hz to 37.1 μA at 6 Hz—because higher operating frequencies shorten the charge–transfer duration and consequently enhance the instantaneous current. In contrast, Figure 3c reveals that the *Q*_SC_ remains essentially constant across the examined frequency range, maintaining a nearly unchanged value of approximately 104.1 nC, which indicates that the total amount of triboelectric charge generated per cycle is predominantly determined by the interfacial contact area and surface charge density rather than the operation speed. In Figure 3d, *V*_OC_ increases as the maximum gap distance between the nylon film and the PAC hydrogel is enlarged from 3 to 11 mm. A larger separation distance produces a higher electrostatic potential difference between the two electrodes, thereby boosting the output voltage. The *I*_SC_ curves in Figure 3e display a similar monotonic growth trend, which can be attributed to the enhanced potential gradient and stronger driving force for electron flow in the external circuit. Figure 3f further confirms that *Q*_SC_ also increases with separation distance, suggesting that a larger effective gap facilitates more complete charge induction and reduces back‐recombination within one cycle, leading to improved charge transfer capability. The effect of the gap distance between the nylon film and the PAC hydrogel on the electrical output performance was investigated in the range of 3–11 mm. This range was selected to ensure stable and repeatable contact‐separation behavior under the current experimental setup. As the gap distance increases, the output voltage, current, and transferred charge show an approximately linear increase within the tested range. This behavior can be attributed to the enhanced electrostatic potential difference and more effective charge separation during the contact‐separation process. It should be noted that this linear trend is valid only within the investigated range, and further increase in gap distance may lead to deviation from linearity or saturation due to limitations in effective electrostatic interaction and charge density. The role of Ca^2+^ concentration in the PVA/Ala/CaCl_2_ hydrogel electrode is investigated in Figure 3g–i. When the CaCl_2_ content is increased from 1 to 10 wt%, *V*_OC_ (Figure 3g) and *I*_SC_ (Figure 3h) first rise and then slightly decline, with the highest values obtained at an intermediate Ca^2+^ loading (6 wt%). The same volcano‐type trend is observed for *Q*_SC_ in Figure 3i. At low Ca^2+^ contents, the introduction of Ca^2+^/Cl^−^ ions significantly enhances ionic conductivity and strengthens ionic cross‐linking, which improves interfacial contact, facilitates charge transport in the hydrogel, and thus elevates the electrical output. However, excessive CaCl_2_ leads to ion crowding and stronger electrostatic screening within the hydrogel network. The shortened Debye length and increased leakage pathways suppress effective charge separation at the triboelectric interface and dissipate part of the induced charges before they can be collected, resulting in reduced *V*_OC_, *I*_SC_, and *Q*_SC_. Therefore, the Ca^2+^ concentration of 6 wt% represents an optimal balance between sufficient mobile ions for conduction and minimal charge‐screening loss, yielding the best overall TENG performance.

### 3.5 Output Characteristics and Energy Harvesting Capability of PAC‐TENG Device

**FIGURE 4:**
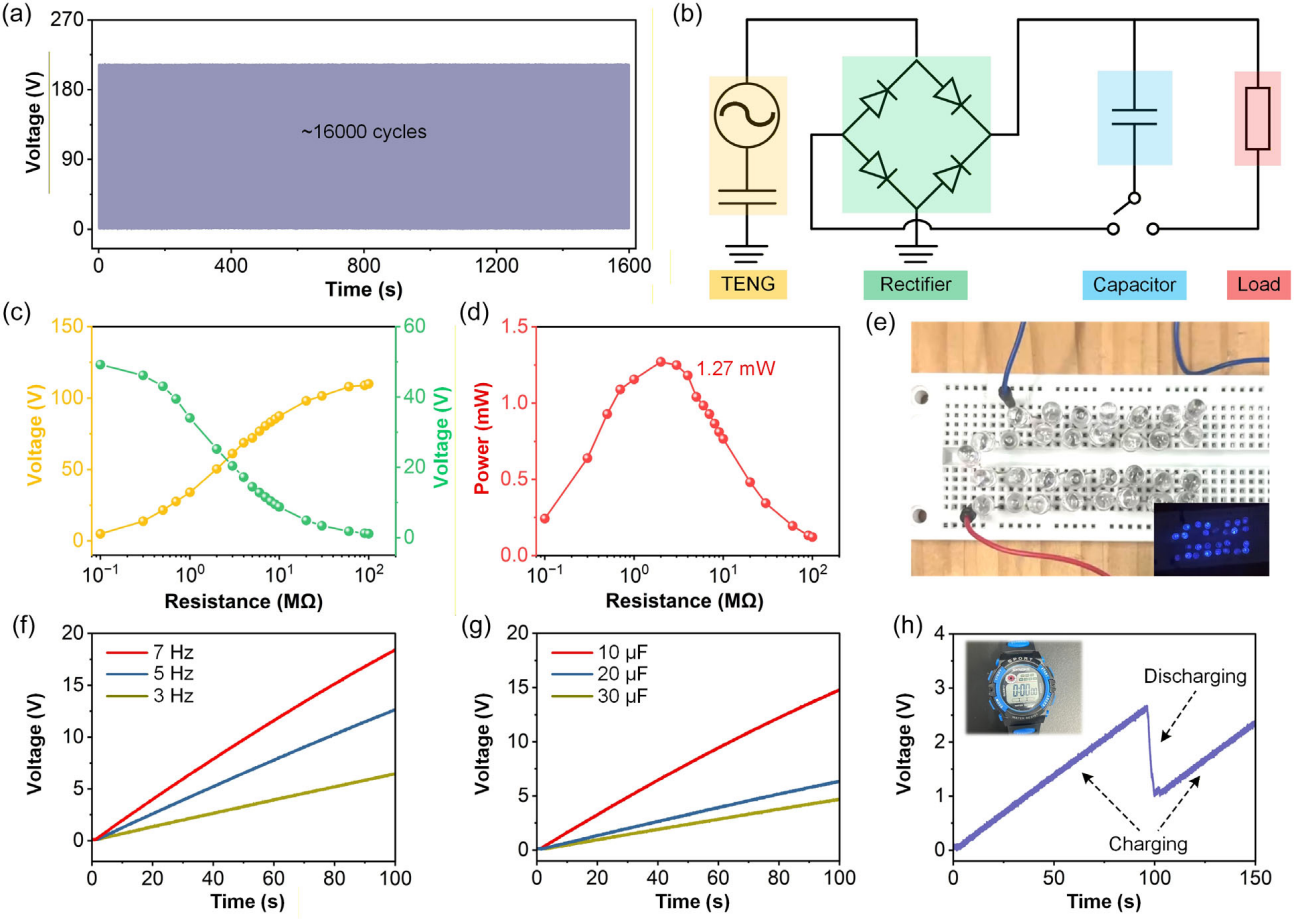
**Output durability, power management, and energy‐storage performance of the PAC‐TENG.** (a) Long‐term durability test of the PAC‐TENG. (b) Schematic diagram of the self‐charging energy‐storage circuit consisting of the PAC‐TENG. (c) Output voltage of the PAC‐TENG as a function of external load resistance. (d) Output power of PAC‐TENG calculated under different load resistances. (e) Photograph of an LED array directly powered by the PAC‐TENG through a bridge‐rectified output. (f) Charging curves of a 10 µF capacitor at different operation frequencies (3, 5, and 7 Hz). (g) Charging behavior of capacitors with different capacitances (10, 20, and 30 µF) at a fixed frequency. (h) Charging and discharging profile of a commercial electronic watch powered by the PAC‐TENG through a 10 µF capacitor.

Figure 4a illustrates the long‐term operational durability of the PAC‐TENG under continuous cyclic excitation. Over approximately 16 000 contact‐separation cycles, the output voltage remains highly stable without observable decay, confirming that the silicone‐encapsulated hydrogel electrode effectively maintains structural integrity and charge–transfer efficiency during extended operation. This durability reflects the synergistic mechanical robustness of the PVA/Ala/CaCl_2_ hydrogel and the compliant silicone layer, which together suppress fatigue‐induced failure and preserve interfacial electrification. To evaluate the capability of the device in practical energy‐harvesting systems, a rectification and storage circuit was constructed, as schematically presented in Figure 4b. The AC output of the PAC‐TENG is converted into DC through a full‐bridge rectifier and subsequently stored in a capacitor before powering external loads. This setup represents a standard self‐charging architecture suitable for wearable and portable electronics. The dependence of output voltage and current on external load resistance is shown in Figure 4c. As resistance increases from sub‐megaohm to tens of megaohms, the voltage steadily rises owing to suppressed charge leakage, while the current exhibits a monotonic decline due to limited charge–transfer rates across higher impedance. The crossover between these two characteristics leads to an optimal power output, as quantified in Figure 4d. The corresponding power curve displays a pronounced peak at approximately 1.27 mW under a load near a few megaohms, demonstrating the efficient energy‐conversion capability of the PAC‐TENG and the suitability of the hydrogel electrode for high‐impedance circuits often found in low‐power electronics. The practical output performance is further demonstrated in Figure 4e, where the PAC‐TENG directly lights an array of commercial LEDs through the rectifier–capacitor system. The continuous and stable emission of multiple LEDs verifies the capability of the device to supply instantaneous high‐voltage pulses as well as sustained DC power following rectification. Figure 4f examines the charging characteristics of a 10 µF capacitor at different operating frequencies. Higher frequencies consistently yield faster charging rates, confirming that increased contact‐separation rates enable more frequent charge transfer and thus higher average power output. Figure 4g further compares capacitors of different capacitances at a fixed frequency. As expected, smaller capacitance values lead to faster voltage accumulation, whereas larger capacitors require longer charging durations but store more energy overall. Figure 4h demonstrates the ability of the PAC‐TENG to charge and power a digital wristwatch. The charging curve shows a gradual voltage rise to above the operational threshold of the watch, followed by a clear discharge stage once the device is activated. This result highlights the feasibility of applying the PAC‐TENG as a sustainable power source for low‐power wearable electronics, reinforcing its potential in self‐powered sensing and portable energy‐harvesting applications.

### 3.6 Self‐Powered Joint Motion Sensing of the PAC‐TENG for Swimming Motion Monitoring

**FIGURE 5:**
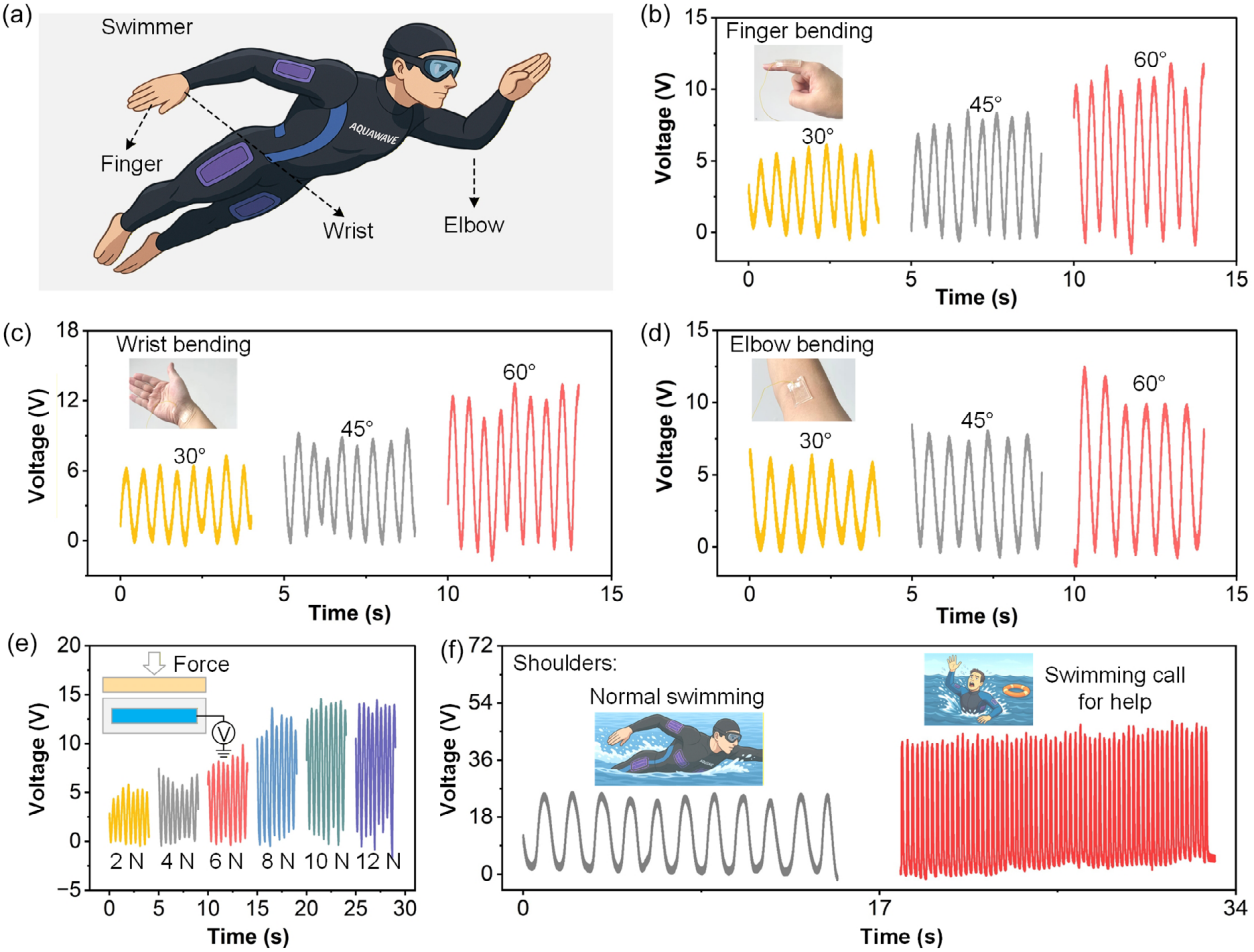
**Overview of the wearable PAC‐TENG for aquatic motion monitoring and emergency signaling.** (a) Schematic illustration showing the placement of PAC‐TENG sensors on a swimmer's finger, wrist, and elbow for real‐time biomechanical monitoring during aquatic activities. (b–d) Output voltage signals generated by PAC‐TENG under controlled bending of the finger, wrist, and elbow at different joint angles (30°, 45°, and 60°). (e) Voltage responses of PAC‐TENG under various applied forces. (f) Distinct signal patterns were produced during normal swimming strokes and simulated emergency arm‐waving motions.

Figure 5a schematically illustrates the deployment scenario, in which multiple PAC‐TENG patches are placed on the swimmer's finger, wrist, and elbow. These positions are chosen because they undergo characteristic angular deformation during each swimming stroke, producing distinct mechanical stimuli that can be effectively captured by the sensor. The illustration also highlights the potential for distributed sensor networks on wetsuits, enabling full‐body aquatic motion mapping. Figure 5b shows the voltage outputs generated during finger bending at 30°, 45°, and 60°, respectively. As the bending angle increases, both the amplitude and density of the output signals increase accordingly. This trend clearly validates the sensor's high angular sensitivity and demonstrates its potential for tracking subtle variations in finger paddling motions, which strongly influence stroke efficiency and water‐entry coordination. Similarly, Figure 5c presents wrist bending responses at the same angles. The wrist is one of the most active joints during freestyle and butterfly strokes, and the PAC‐TENG outputs show progressively amplified waveforms with increasing angle. The nearly sinusoidal patterns further confirm excellent mechanical adaptability and stability, suggesting that the sensor can reliably differentiate wrist‐rotation amplitudes originating from different swimming styles. Figure 5d displays elbow bending outputs, again at 30°, 45°, and 60°. The elbow joint governs the pulling phase of each stroke cycle, and the recorded signals exhibit distinct voltage enhancement with angle increases. These results collectively confirm that the PAC‐TENG can map multijoint kinematics simultaneously, an essential prerequisite for advanced aquatic‐training analytics or swimming‐posture correction systems. Figure 5e investigates the PAC‐TENG response under varying external forces (2–12 N). Higher forces produce correspondingly stronger voltage amplitudes, demonstrating their capability to quantify underwater propulsion strength or arm‐pull intensity. This function has important implications for evaluating athlete technique, fatigue status, or rehabilitative training progress. Most notably, Figure 5f illustrates a compelling safety‐oriented application. During normal swimming, the shoulder‐mounted PAC‐TENG outputs periodic, moderate‐amplitude signals that reflect regular arm‐swing cycles. In contrast, when simulating an emergency “call‐for‐help” action, the sensor generates significantly denser and more intense pulses due to rapid repeated arm movements. The stark difference between normal and distress patterns indicates that PAC‐TENG‐based wearables could act as self‐powered drowning‐alarm systems, continuously monitoring motion irregularities in real time without requiring external power sources.

## 4 Conclusions

In summary, a highly deformable and environmentally adaptive PAC‐TENG was successfully developed by integrating a PVA/Ala/CaCl_2_ ionic hydrogel electrode with a compliant silicone triboelectric interface. The optimized PAC‐2 hydrogel demonstrated exceptional mechanical resilience, achieving an elongation exceeding 2000%, a tensile strength of 0.41 MPa, and an ionic conductivity up to 0.32 S/m, ensuring stable signal transmission under large deformations. Owing to the synergistic coordination network and effective electron‐accepting silicone surface, the PAC‐TENG delivered excellent electrical performance, producing a peak *V*_OC_ of 472.9 V, an *I*_SC_ of 37.1 µA, and a *Q*_SC_ of 104.1 nC, along with a maximum output power of 1.27 mW under matched load. The device maintained durable cycling stability over 16 000 continuous operation cycles and demonstrated reliable energy‐storage capability by efficiently charging capacitors of different capacitances. Furthermore, the PAC‐TENG exhibited high sensitivity to mechanical variations, enabling accurate detection of joint bending angles, applied forces, and motion frequencies. Its reliable performance in underwater scenarios, including the ability to distinguish swimming stroke patterns and identify irregular motion signals, highlights its strong potential for self‐powered aquatic sports monitoring, wearable biomechanics analysis, and intelligent safety alert systems.

## Conflicts of Interest

The authors declare no conflicts of interest

## Data Availability

Data available on request from the authors.
